# Evaluation of the Anticancer Effects of *Warburgia salutaris* Leaf Extracts: A Comparative Study of Both Liposomal-Encapsulated and Unencapsulated Extracts, with Mechanistic Insights into Apoptotic Signalling

**DOI:** 10.3390/ijms27083567

**Published:** 2026-04-16

**Authors:** Daniel M. Tswaledi, Matlou P. Mokgotho, Makgwale S. Mphahlele, Raymond T. Makola, Jean B. Ngilirabanga, Bwalya A. Witika, Emelinah H. Mathe, Stanley S. Gololo, Ananias H. Kgopa, Leshweni J. Shai

**Affiliations:** 1 Department of Biochemistry and Biotechnology, School of Science and Technology, Sefako Makgatho Health Sciences University, Pretoria 0204, South Africa; lefatswaledi@gmail.com (D.M.T.); emelinah.mathe@smu.ac.za (E.H.M.); stanley.gololo@smu.ac.za (S.S.G.); ananias.kgopa@smu.ac.za (A.H.K.); 2 Department of Physiology, School of Medicine, Sefako Makgatho Health Sciences University, Pretoria 0204, South Africa; 3 Department of Biomedical Sciences, Tshwane University of Technology, Pretoria 0183, South Africa; mphahlelems@tut.ac.za; 4 Department of Biochemistry and Microbiology, University of Limpopo, Sovenga 0727, South Africa; raymond.makola@ul.ac.za; 5 Department of Pharmaceutical Sciences, School of Pharmacy, Sefako Makgatho Health Sciences University, Pretoria 0204, South Africa; jean.ngilirabanga@smu.ac.za (J.B.N.); bwalya.witika@smu.ac.za (B.A.W.)

**Keywords:** cancer, *Warburgia salutaris*, liposomes, cellular metabolic activity, apoptosis

## Abstract

Although medicinal plants possess vast biological properties, crude medicinal plant extracts often show limited therapeutic efficacy due to poor aqueous solubility, instability, and inadequate bioavailability, which restricts efficient intracellular delivery. As cancer is a genetic disease requiring intracellular and nuclear targeting, improved delivery systems are essential. *Warburgia salutaris* is traditionally used in Southern Africa and possesses reported anticancer and anti-inflammatory properties; however, its crude extracts exhibit suboptimal delivery characteristics. This study comparatively evaluated the anticancer effects of unencapsulated (WSN) and liposomal-encapsulated (WSE) crude leaf extracts, with emphasis on apoptotic mechanisms. Liposomal formulation was confirmed by FTIR, PXRD, and DLS, yielding stable nanoparticles (159.4 nm; PDI 0.114; +79.3 mV). Both WSN and WSE demonstrated efficacy and concentration-dependent cytotoxicity against MCF-7 breast cancer cells (IC_50_ < 0.0195 mg/mL) with minimal toxicity toward Vero kidney cells and RAW 264.7 macrophages. Mechanistically, WSN induced rapid cytotoxicity with necrotic features, whereas WSE promoted regulated apoptosis. Apoptosis was validated by DAPI/PI staining, Annexin V/PI flow cytometry, mRNA expression levels of Bax, Bcl-2, and caspase-3 measured with RT-PCR and proteome profiling array, confirming activation of intrinsic and extrinsic pathways. Both extracts also reduced LPS-induced ROS production. LC-MS identified multiple bioactive phytochemicals. Overall, liposomal encapsulation enhanced therapeutic precision, stability, and selectivity cytotoxicity, supporting its development as a nanomedicine-based anticancer strategy.

## 1. Introduction

Cancer is a non-communicable disease associated with significant morbidity and mortality worldwide. It is characterised by uncontrolled cell proliferation, metastatic progression, and resistance to apoptosis [1,2]. Despite advances in chemotherapy, immunotherapy, and targeted molecular therapies, effective cancer treatment remains limited by poor tumour selectivity, systemic toxicity, therapeutic resistance and inadequate bioavailability of many anticancer agents [3,4,5]. These challenges are particularly pronounced in low- and middle-income regions, including sub-Saharan Africa, where delayed diagnosis and limited access to treatment contribute significantly to cancer-related mortality [6,7].

Natural products and plant-derived compounds continue to play a central role in anticancer drug discovery and development, providing structurally diverse bioactive molecules capable of modulating multiple cancer-associated pathways [6,7,8]. Several clinically used anticancer agents have originated from medicinal plants, highlighting the therapeutic relevance of phytochemicals [9,10]. Plant-derived compounds have been shown to regulate key cellular processes, including cell-cycle arrest, oxidative stress modulation, mitochondrial dysfunction and induction of apoptosis [11,12,13]. Despite promising in vitro activity, many crude plant extracts exhibit limited clinical translation due to poor aqueous solubility, instability, and inefficient intracellular delivery [14,15].

*Warburgia salutaris* (Canellaceae), commonly known as the pepper-bark tree, is an indigenous Southern African medicinal plant widely used in traditional medicine for the treatment of inflammation, infections, and tumour-related conditions [16]. *Warburgia salutaris* (Bertol. f.) Chiov. is an aromatic evergreen tree native to southern Africa, particularly distributed across regions such as Limpopo, Mpumalanga, and KwaZulu-Natal. The species typically grows between 5 and 20 m in height and is characterised by smooth grey bark and glossy, aromatic leaves. Various parts of the plant, including the bark, leaves, and root bark, are highly valued for both medicinal and aromatic purposes, playing a central role in traditional healthcare systems [17,18,19].

Traditionally, *W. salutaris* has been extensively utilised by indigenous communities for the treatment of a wide spectrum of ailments, with more than 20 ethnomedicinal applications reported. These include the management of respiratory conditions such as bronchitis and chest infections, gastrointestinal disorders including ulcers, infectious diseases such as malaria and thrush, as well as inflammatory conditions, fever, rheumatic pain, and venereal diseases. Notably, among the Zulu community, the plant has been specifically used in the management of cancer, highlighting its ethnopharmacological relevance in oncology. In addition to its medicinal applications, *W. salutaris* is also associated with cultural and spiritual practices, where plant materials are used in traditional healing rituals [17,18,19,20].

Phytochemical investigations have revealed that *W. salutaris* contains a diverse array of bioactive compounds, particularly drimane-type sesquiterpenoids such as warburganal, polygodial, muzizadial, mukaadial, and salutarisolide, alongside polyphenols and other terpenoid compounds. These constituents exhibit antibacterial, antioxidant, anti-inflammatory, and cytotoxic activities [21,22]. Importantly, several of these phytoconstituents have been shown to exert selective cytotoxic effects against cancer cells through mechanisms involving oxidative stress modulation, interaction with thiol-containing proteins, disruption of mitochondrial membrane potential, and activation of apoptotic signalling pathways. The induction of apoptosis via reactive oxygen species (ROS) generation is considered a central mechanism underlying the anticancer activity of *W. salutaris* [23,24].

Scientific studies have further validated several traditional claims, demonstrating that isolated compounds such as muzizadial exhibit cytotoxic, antibacterial, and anticancer properties. However, despite these promising findings, the clinical translation of *W. salutaris* remains limited due to insufficient pharmacokinetic data, including absorption, distribution, metabolism, and excretion (ADME), as well as concerns related to toxicity and poor aqueous solubility of crude extracts [25,26,27]. These limitations highlight the need for advanced drug delivery strategies to improve bioavailability, enhance target specificity, and maximise therapeutic efficacy.

*Warburgia salutaris* phytochemicals analysis has demonstrated a plethora of bioactive compounds, including sesquiterpenes, flavonoids, lignans, diarylheptanoids, and related secondary metabolites with reported cytotoxic, anticancer, and anti-inflammatory properties [28,29,30,31]. Previous studies have demonstrated that extracts of *W. salutaris* exhibit significant cytotoxic effects against various cancer cell lines. However, these effects are often accompanied by limited selectivity and non-specific cytotoxicity when administered as crude formulations [32,33].

Nanotechnology-based drug delivery systems have emerged as promising tools to overcome the limitations associated with conventional phytochemical delivery by improving solubility, stability, and intracellular uptake [34,35,36]. Among these systems, liposomes are particularly attractive due to their biocompatibility and ability to encapsulate both hydrophilic and hydrophobic compounds. Moreover, they are capacity to enhance therapeutic potency while reducing systemic toxicity [37,38,39]. Importantly, liposomal encapsulation has been shown to modulate cancer cell death pathways, shifting cytotoxic responses from immune-triggering necrosis toward regulated apoptosis, thereby overcoming inflammation and off-target tissue damage [40,41,42].

Apoptosis is a highly regulated form of programmed cell death that plays a critical role in maintaining tissue homeostasis and eliminating damaged or malignant cells [43,44]. Dysregulation of apoptotic signalling pathways is a cancer pathogenesis leading to tumour progression and therapeutic resistance [45,46]. Apoptotic cell death may be initiated through intrinsic (mitochondrial-mediated) or extrinsic (death receptor-mediated) pathways, both of which converge on the activation of executioner caspases such as caspase-3 [24,47,48]. Therapeutic strategies that restore or enhance apoptotic signalling are therefore considered central to effective anticancer intervention [49,50].

In addition to apoptosis resistance, chronic inflammation and excessive reactive oxygen species (ROS) production contribute to tumour initiation, progression, and metastasis [51,52]. During an inflammatory response, macrophages elicit ROS production, which can promote a tumour-supportive microenvironment [53]. Consequently, agents capable of simultaneously inducing apoptosis in cancer cells while suppressing inflammation-associated ROS production may offer enhanced therapeutic benefit [54,55]. Despite the documented biological activity of *W. salutaris*, the impact of the nanocarrier on drug delivery in an attempt to enhance anticancer potency is underexplored [56].

Therefore, the present study aimed to comparatively evaluate the anticancer effects of unencapsulated and liposomal-encapsulated *W. salutaris* crude leaf extracts, with specific emphasis on apoptotic pathway activation, selective cytotoxicity and oxidative stress modulation. Crude extracts were prepared using a dichloromethane-methanol solvent system to facilitate the extraction of both non-polar and moderately polar phytochemicals

## 2. Results

### 2.1. Phytochemical Characteristics Analysed by FTIR

**Figure 1:**
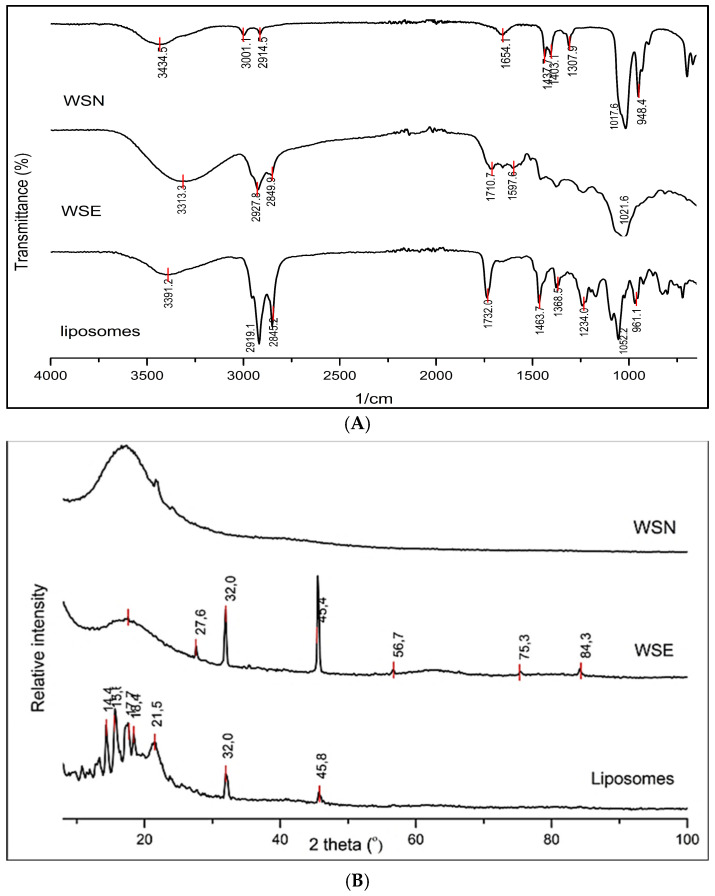
(**A**) Displays the FTIR spectra of *W. salutaris* unencapsulated (WSN), liposomal-encapsulated (WSE) crude leaf extracts and liposomes. (**B**) Presents the Powder X-ray diffraction (pXRD) analysis of the three samples mentioned above.

FTIR spectroscopy was used to characterise functional groups and assess interactions between the *W. salutaris* crude extract, blank liposomes, and encapsulated liposomes (Figure 1A). The crude extract exhibited a broad O–H stretching band centred at ~3300 cm^−1^, consistent with hydroxyl groups of phenolic constituents and residual moisture. A moderate absorption band near 1680 cm^−1^ corresponded to C=O stretching of aldehydes and ketones, while signals within 1450–1600 cm^−1^ were attributed to aromatic C=C stretching vibrations. Prominent C–O stretching bands between 1000 and 1300 cm^−1^ indicated the presence of alcohols and ether functionalities typical of phytochemicals.

The blank liposomes displayed characteristic lipid-associated bands, including aliphatic C–H stretching (2850–2950 cm^−1^), ester C=O stretching (1735 cm^−1^), and phosphate group vibrations at 1230 cm^−1^ (P=O) and 1080 cm^−1^ (P–O–C). The encapsulated formulation exhibited combined spectral features of both the extract and lipid components. The persistence of the O–H band and slight shifts and broadening of C=O and phosphate peaks relative to blank liposomes indicate molecular interactions between extract constituents and the lipid bilayer.

### 2.2. Powder X-Ray Diffraction (PXRD)

PXRD analysis was conducted to evaluate structural characteristics (Figure 1B). The crude extract showed a broad diffuse halo centred around 20° 2θ, confirming its amorphous nature. Blank liposomes composed of DPPC and cholesterol exhibited sharp diffraction peaks between 10° and 40° 2θ, indicative of a semi-crystalline lipid bilayer structure. The encapsulated formulation displayed a superimposed pattern comprising the amorphous halo of the extract and the crystalline reflections of the lipid matrix, demonstrating successful incorporation of the extract without disruption of lipid crystallinity.

### 2.3. Particle Size and Zeta Potential

Dynamic light scattering analysis (Table 1) revealed that the optimised encapsulated formulation had a mean particle size of 159.4 nm with a narrow size distribution (PDI = 0.114). The liposomes exhibited a high positive zeta potential of +79.3 mV. These parameters confirm the formation of nanosized, monodisperse vesicles with strong electrostatic stability, supporting colloidal integrity and resistance to aggregation.

### 2.4. MTT Assay

**Figure 2:**
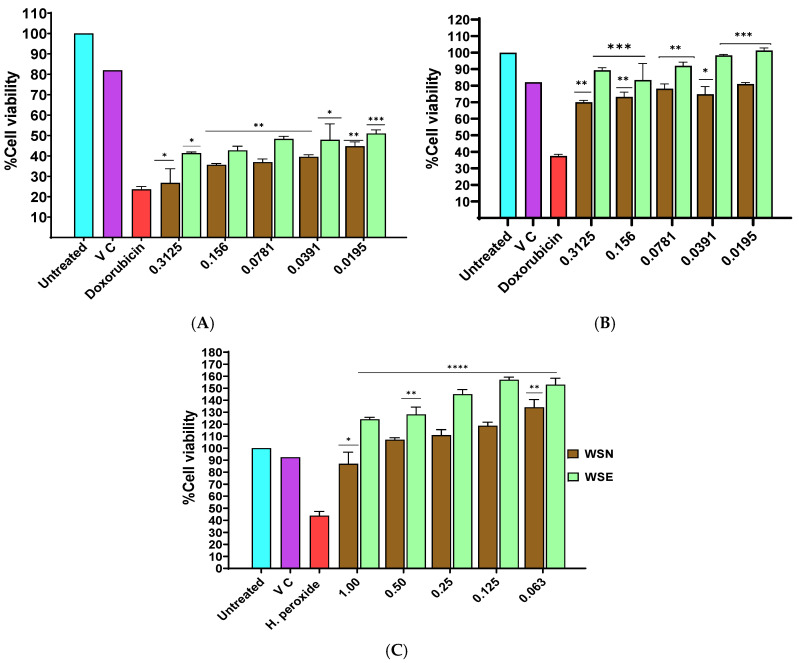
Cytotoxic effects of WSN and WSE extracts using the MTT assay. The cytotoxicity was evaluated after 24 h of treatment with WSN and WSE extracts. The MCF-7 (**A**) and Vero (**B**) cells were treated with different concentrations ranging from 0.3125 to 0.0195 mg/mL, and doxorubicin served as the positive control. RAW 264.7 cells (**C**) were treated with concentrations ranging from 1 to 0.063 mg/mL, with hydrogen peroxide used as a positive control, showing no detectable cytotoxicity with cell viability remaining above 100% and IC_50_ values not reached within the tested range. Untreated cells served as the negative control, and DMSO was used as the vehicle control (VC). Data represent the mean ± standard deviation of two independent experiments, with * *p* ≤ 0.05, ** *p* ≤ 0.01, *** *p* ≤ 0.001, and **** *p* ≤ 0.0001 indicating significant differences compared to the control group.

The cytotoxic effects of WSN and WSE were evaluated using the MTT viability assay following 24 h exposure on MCF-7 breast cancer cells, Vero kidney epithelial cells, and RAW 264.7 macrophages (Figure 2). In MCF-7 cells (A), both extracts induced a significant, concentration-dependent reduction in metabolic activity across 0.3125–0.0195 mg/mL relative to untreated controls. Cell viability declined below 50% within this range, with IC_50_ values < 0.0195 mg/mL. The magnitude of cytotoxicity was comparable to the positive control, doxorubicin, which similarly produced a marked decrease in cell viability. In Vero cells (B), WSN and WSE demonstrated substantially lower cytotoxicity. IC_50_ values were markedly higher than those observed in MCF-7 cells, and cell viability remained above 75% across all tested concentrations. Statistical analysis revealed only minor differences compared with untreated controls. In contrast, doxorubicin significantly reduced metabolic activity in Vero cells (*** *p* ≤ 0.001). In RAW 264.7 macrophages (C), exposure to WSN and WSE (1–0.063 mg/mL) did not significantly alter metabolic activity relative to controls, and no IC_50_ values were reached within the tested range. Conversely, hydrogen peroxide significantly decreased cell viability (**** *p* ≤ 0.0001), confirming assay sensitivity. Collectively, these data demonstrate potent and selective cytotoxicity of WSN and WSE toward MCF-7 cells, with minimal effects on non-cancerous Vero cells and immune-derived RAW 264.7 macrophages.

### 2.5. Anti-Metastatic Effect on MCF-7 Cells Treated with WSN and WSE Leaf Extracts

**Figure 3:**
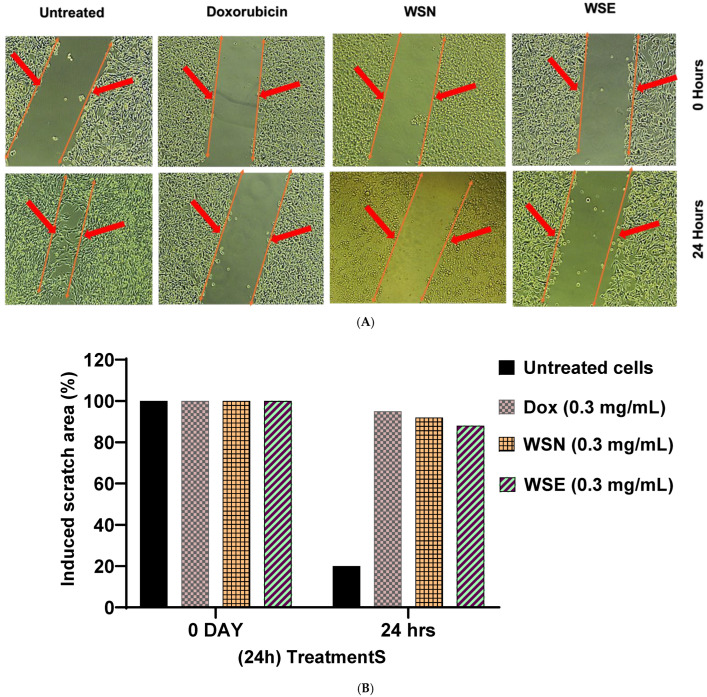
(**A**) Scratch assay images demonstrating anti-metastatic effects (arrows indicating cell migration) and (**B**) a graphical representation of the percentage of the scratched area in MCF-7 cells after treatment with WSN and WSE extracts, evaluated after 24 h of exposure. Doxorubicin served as a positive control, while untreated cells acted as a negative control.

The antimetastatic potential of WSN and WSE was evaluated using a scratch assay in MCF-7 cells (Figure 3). Representative micrographs captured at 0 and 24 h following treatment are shown in Figure 3A. Cells were treated with 0.3 mg/mL of WSN, WSE, or doxorubicin, with untreated cells serving as the negative control and doxorubicin as the positive control. At 24 h, untreated cells exhibited partial closure of the scratch area, indicating active cell migration. In contrast, treatment with doxorubicin markedly inhibited wound closure, confirming suppression of migratory capacity. Similarly, both WSN- and WSE-treated cells demonstrated significant inhibition of scratch closure compared to the untreated control. The wound area remained largely open after 24 h, reflecting impaired cell migration.

Quantitative analysis of the scratch area (Figure 3B) corroborated the microscopic observations. All treated groups showed a significant reduction in wound closure relative to untreated cells, indicating inhibition of migratory behaviour. The extent of migration inhibition observed with WSN and WSE was comparable to that of doxorubicin under the experimental conditions.

These findings demonstrate that both unencapsulated and liposome-encapsulated *W. salutaris* crude leaf extracts effectively suppress MCF-7 cell migration in vitro.

### 2.6. Determination of W. salutaris-Induced Apoptotic Features Using DAPI/PI Staining

**Figure 4:**
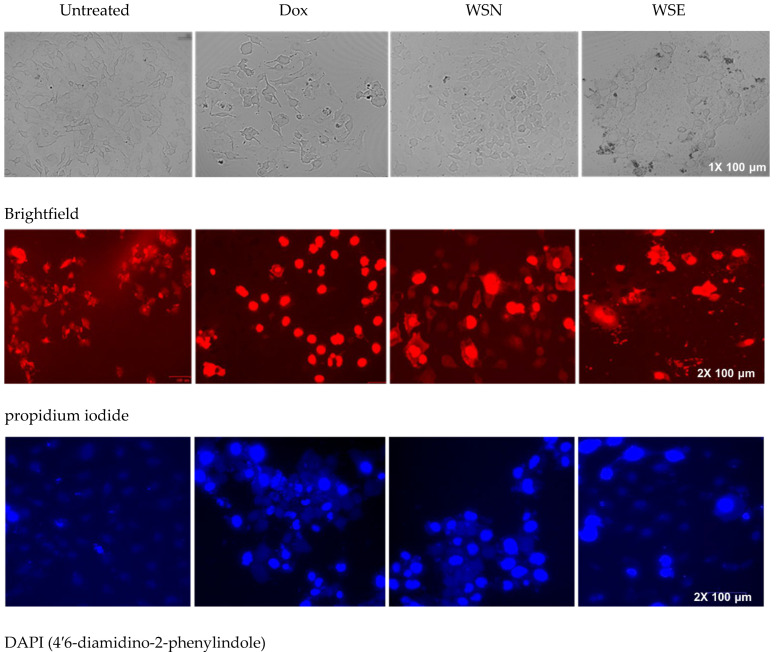
Brightfield and fluorescent images of MCF-7 cells following a 24-h incubation with 0.3 mg/mL crude unencapsulated WSN and liposomal-encapsulated WSE leaf extracts of *W. salutaris*. Doxorubicin hydrochloride was used as the positive control, while untreated cells served as the negative control. Images were captured using a Zoe Fluorescent Cell Imager (Bio-Rad Laboratories, Hercules, CA, USA). Magnification/zoom levels are indicated as follows: brightfield images, 1× (scale bar = 100 µm); PI and DAPI fluorescent images, 2× (scale bar = 100 µm).

Induced mode of cell death on MCF-7 cells was evaluated using dual DAPI/PI staining following treatment with WSN, WSE (0.3 mg/mL), and doxorubicin (positive control) (Figure 4). Untreated cells exhibited intact nuclei with uniform DAPI staining and slight PI uptake, indicating preserved membrane integrity. Doxorubicin showed intense DAPI fluorescence with marked nuclear condensation, accompanied by strong PI staining, confirming late apoptosis. Cells treated with WSN and WSE extracts exhibited nuclear condensation and chromatin margination, which were confirmed by stronger DAPI fluorescence signals compared with the control samples. PI-positive cells were observed in both treatment groups, confirming membrane permeabilisation. Fluorescence intensity analysis indicated apoptotic induction in both groups, with WSN displaying stronger overall fluorescence relative to WSE. Brightfield microscopy further revealed morphological alterations in treated cells, including reduced cell density, cellular shrinkage, and structural disintegration, consistent with apoptotic progression. WSN and WSE induce nuclear condensation, chromatin margination, and membrane permeabilisation in MCF-7 cells, confirming apoptosis, with WSN showing slightly stronger effects than WSE.

### 2.7. Apoptotic Gene Expression Levels in MCF-7 Cells Analysed by RT-PCR

**Figure 5:**
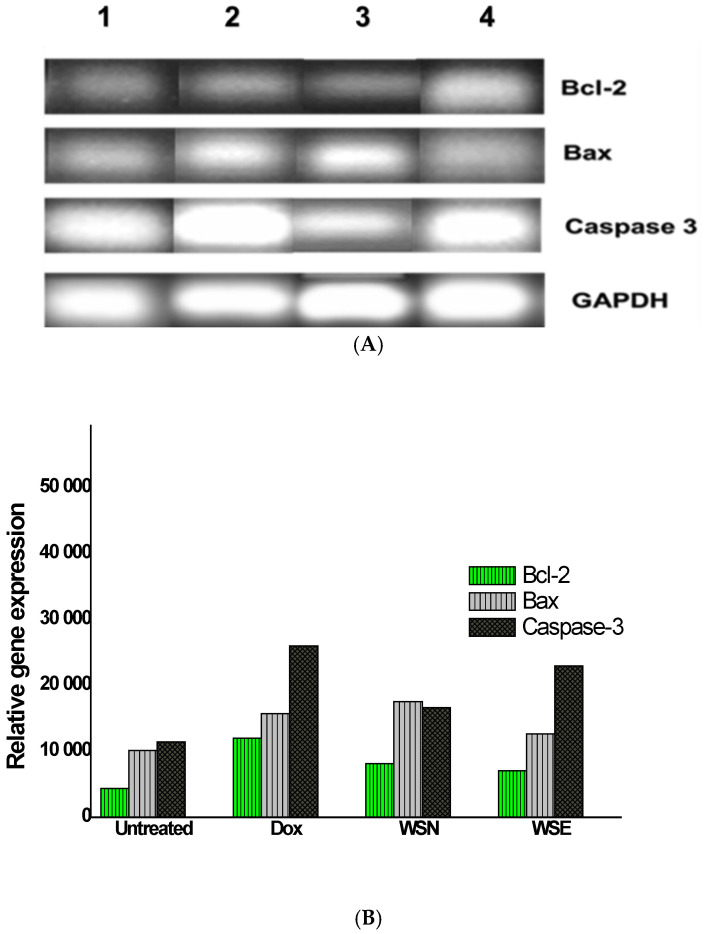
(**A**) Bands depicting expression levels of the apoptosis-related genes Bax, *Bcl-2*, *caspase-3*, and *GAPDH* were analysed after amplification using PCR. Gel electrophoresis was performed, producing the bands, and Zeo fluorescent microscopes (Bio-Rad Laboratories, Hercules, CA, USA) were used to visualise them. The legend in the images indicates: 1: untreated control, 2: Doxorubicin as a positive control, 3: WSN, and 4: WSE crude leaf extracts. (**B**) The graph illustrates the percentage band intensity of the expression levels of apoptosis-associated genes. The data represent the mean and standard deviation of the experiment. A *p*-value below 0.05 indicates that the results are statistically different from the negative control (untreated).

Reverse Transcription (RT-PCR) was used to evaluate gene expression on MCF-7 cells post exposure to *W. salutaris*, focusing specifically on pro-apoptotic and anti-apoptotic genes that include *Bax*, *Bcl-2*, and *caspase 3*. The *GAPDH* served as the reference gene. Gel electrophoresis was used to visualise the PCR amplicons. Figure 5A depicts the expressed bands alongside Figure 5B, which was used to illustrate the expression levels of each gene graphically. The findings indicate that the pro-apoptotic gene *Bax* increased the expression of *caspase 3*, while *Bcl-2* was downregulated following treatment. Figure 5B graphically supported the data, showing high percentages of the pro-apoptotic gene expression compared to anti-apoptotic genes in both the WSN and WSE.

### 2.8. Analysis of the Human Apoptotic Proteome Profiling

**Figure 6:**
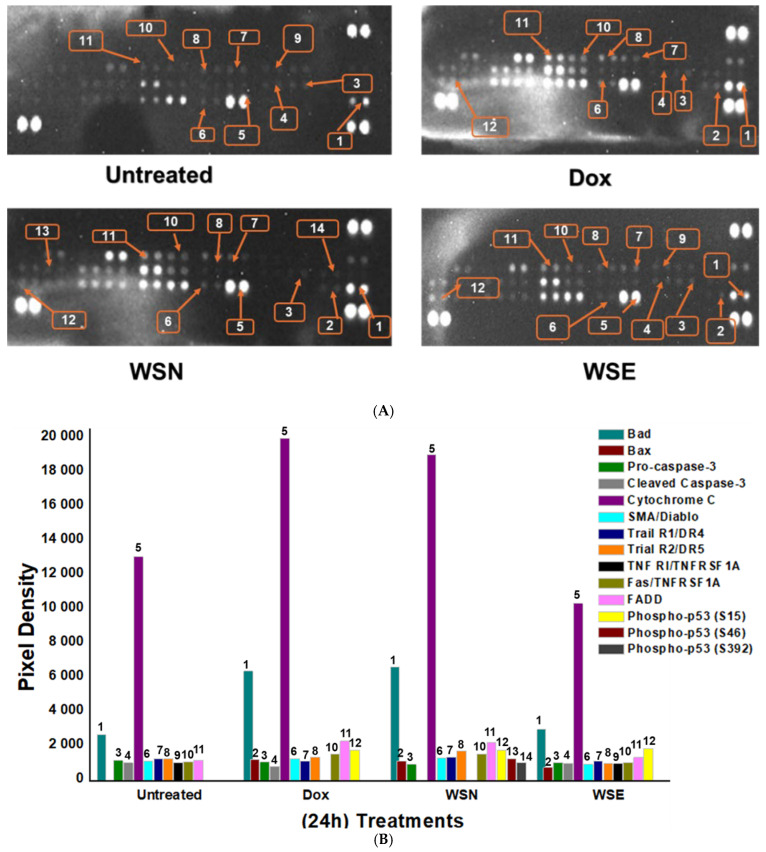
Images of pixel density produced by human apoptotic proteins expressed in MCF-7 cells. Doxorubicin hydrochloride served as the positive control, while untreated cells acted as the negative control. A total of 14 pro-apoptosis proteins were observed using, (**A**) ChemiDoc XRS+ Visualiser (Bio-Rad Laboratories (Pty) Ltd., Johannesburg, South Africa) and (**B**) the percentage intensity of the pixels produced.

The human apoptotic proteome profiler detected multiple proteins associated with apoptosis, indicating that apoptosis was activated in MCF-7 cells treated with 0.3 mg/mL of unencapsulated WSN and liposomal-encapsulated WSE leaf extracts through protein profiling. Both extracts increased the expression of *Bax*, *Bad*, *cleaved caspase-3*, *SMAC/Diablo*, *TRAIL R1/DR4*, *TRAIL R2/DR5*, *TNF RI*, *Fas/CD95*, *FADD*, and *phosphorylated p53 (S15*, *S46*, *S392)*, while reducing *Bcl-2* and *pro-caspase-3* levels. Notably, both extracts markedly induced cytochrome c release, representing a key event in mitochondrial-mediated apoptosis (Figure 6A). The pixel density of the expressed apoptotic proteins was shown in Figure 6B), supporting the indication of the bend intensity. In contrast, doxorubicin did not exhibit comparable cytochrome c expression under the tested conditions. WSN and WSE trigger both intrinsic (mitochondrial) and extrinsic apoptotic pathways, as evidenced by cytochrome c release and activation of key apoptotic proteins.

### 2.9. Analysis of Apoptosis Profile in MCF-7 Using the Annexin V/PI Staining Method

**Figure 7:**
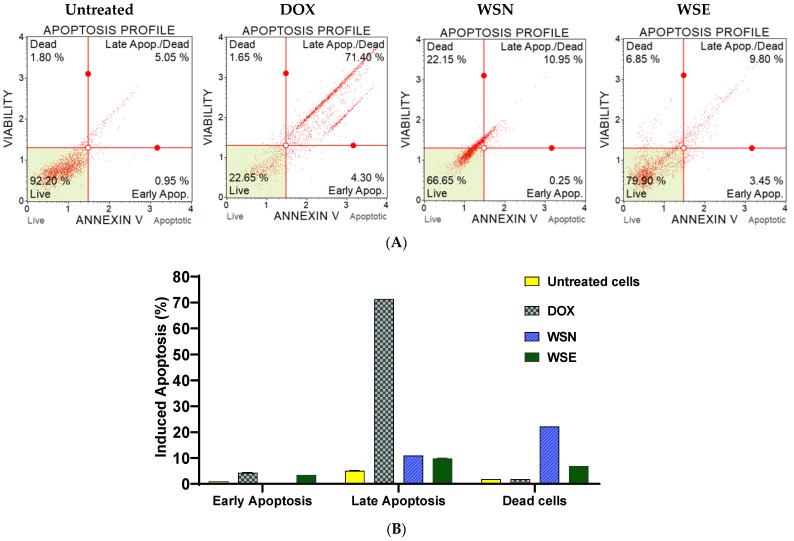
(**A**) displays images showing the effects of treatments on MCF-7 cells by inducing a type of cell death at a dose of 0.3 mg/mL of unencapsulated WSN and liposomal-encapsulated WSE leaf extracts. Doxorubicin hydrochloride served as the positive control, while untreated cells acted as the negative control. Muse^®^ Cell (Luminex Corporation, Austin, TX, USA) Analyse was used to analyse the treated cells, which were categorised into four quadrants: (1) live, (2) early apoptosis, (3) late apoptosis, and (4) dead cells. (**B**) Shows the percentage of cell death.

Figure 7A shows the analysis of Annexin V/PI staining using the Muse^®^ Cell Analyser (Luminex Corporation, Austin, TX, USA), confirming that treating MCF-7 cells with unencapsulated (WSN) and liposomal-encapsulated (WSE) crude leaf extracts at 0.3 mg/mL for 24 h induced increased late apoptosis. Untreated cells served as a negative control, while doxorubicin acted as a positive control, showing the highest activation of late apoptosis. Both WSN and WSE extracts resulted in low levels of apoptosis. The WSN extract showed robust cell death compared to the apoptosis process. The WSE extract induced apoptosis, as indicated in the late apoptosis quadrant. Figure 7B highlights that apoptosis was activated in a controlled manner, and late apoptosis showed higher values than early apoptosis. Liposomal encapsulation in WSE favours regulated apoptotic cell death, whereas free WSN indicated direct cell killing, which can lead to necrosis, highlighting the benefit of controlled delivery for therapeutic purposes.

### 2.10. The Effect of WSN and WSE Crude Leaf Extract on ROS Production Measured with H_2_DCF-DA (ROS Probe)

**Figure 8:**
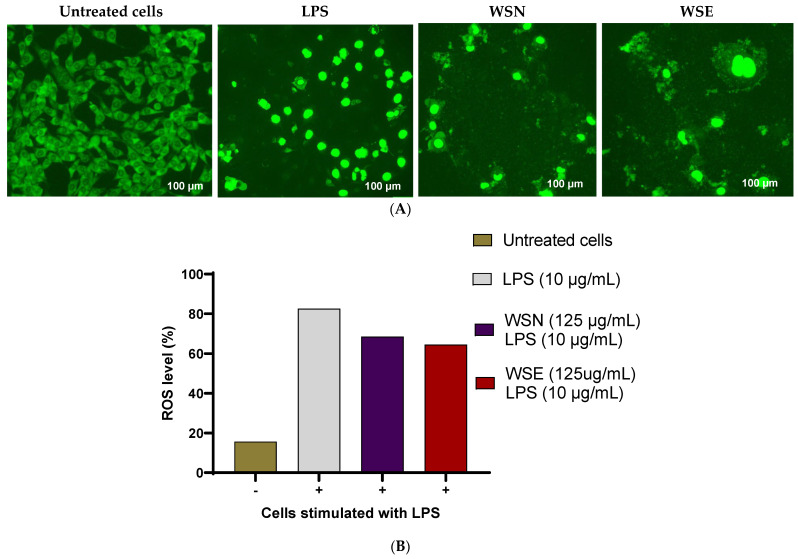
(**A**) The H2DCF-DA assay displays images illustrating the suppression of LPS-induced ROS after treatment. (**B**) The graph shows that cells treated with LPS exhibited significant green fluorescence, indicating increased ROS levels compared to untreated controls. The images depict the effect of LPS-induced ROS generation by extracts, captured using Zoe fluorescent microscopy with the H2DCF-DA dye.

The H_2_DCF-DA assay was used to measure reactive oxygen species (ROS) production induced by lipopolysaccharide (LPS) in RAW 264.7 macrophages treated with unencapsulated (WSN) and liposomal-encapsulated (WSE) crude leaf extracts at a concentration of 0.125 mg/mL. This assay relies on the reaction of H_2_DCF with ROS, which produces fluorescent DCF. The fluorescence intensity correlates with the amount of ROS generated. The Zoe fluorescence images, shown in Figure 8A, confirmed these results, demonstrating that LPS stimulated some cells to emit green fluorescence in both WSN and WSE treatments. However, imaging of WSE indicated a slightly significant reduction compared to the WSN. Both extracts showed the potential of inhibiting LPS-induced ROS production. Figure 8B presents the results graphically, indicating that cells treated with LPS exhibited significant green fluorescence, reflecting increased ROS levels compared to untreated controls. LPS significantly raised ROS production to over 82% relative to controls, demonstrating notable oxidative stress. Both WSN and WSE crude leaf extracts markedly reduced ROS levels, with WSN-treated cells showing approximately a 68% decrease and WSE-treated cells around 64%.

### 2.11. Liquid Chromatography-Mass Spectrometry (LC-MS)

**Figure 9:**
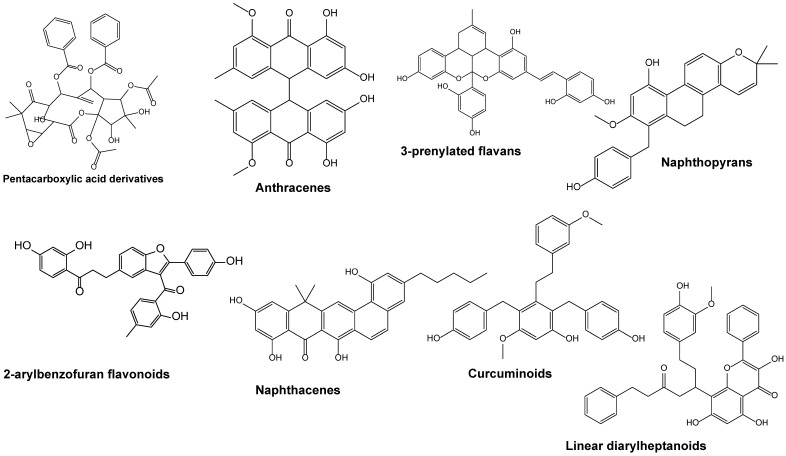

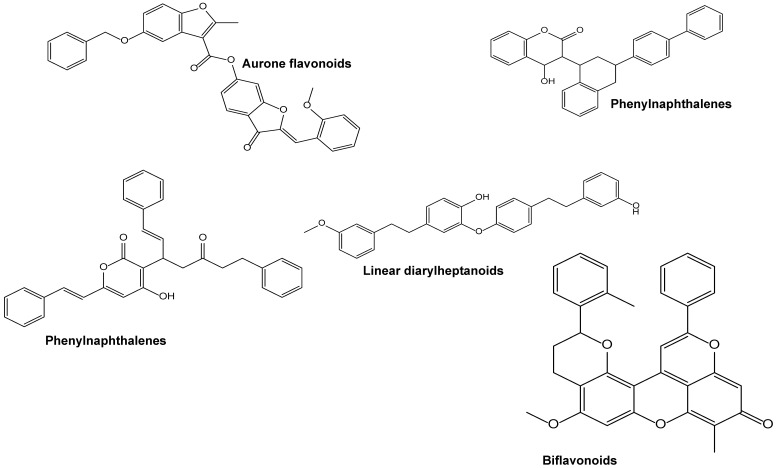

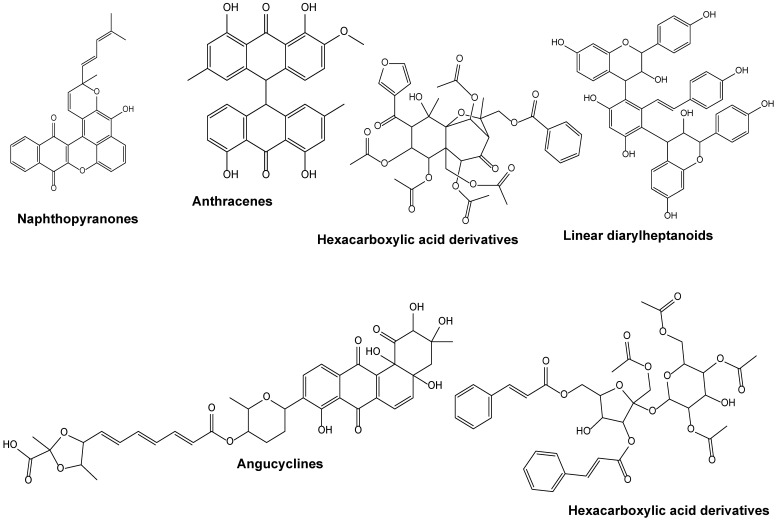

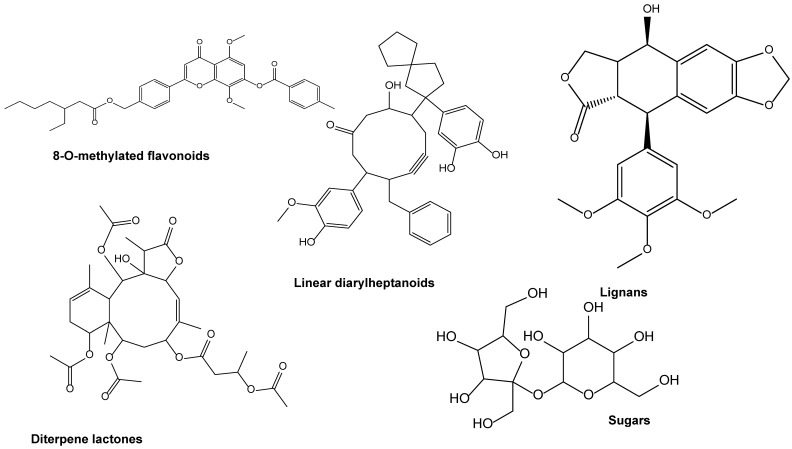
The LC-MS phytochemical analysis displays the structures of biologically active compounds identified in *W. salutaris* crude leaf extracts. ChemDraw Ultra version 12.0 was used to illustrate the structures.

The LC-MS chromatogram of the crude leaf extract of *W. salutaris* prepared with equal parts of dichloromethane and methanol (50:50) displays a broad spectrum of secondary metabolites eluting between 4.47 and 13.94 min. Table 2 lists the twenty-four compounds identified based on their retention time (Rt), mass-to-charge ratio (*m*/*z*), chemical formula, and biological activities. Figure 9 shows the phytochemicals identified in the plant, including flavonoids (such as aurones, prenylated flavans, 8-O-methylated flavonoids, and flavonoid glycosides), lignans, biflavonoids, diarylheptanoids, anthracenes, naphthacenes, naphthopyrans, and curcuminoids, among others. Many of these compounds are associated with established anticancer, cytotoxic, and anti-inflammatory activities (Table 2). The LC-MS profile demonstrates that *W. salutaris* contains a chemically diverse array of bioactive compounds, supporting its observed apoptotic, antioxidant, and anti-inflammatory activities.

## 3. Discussion

This study systematically compared the therapeutic efficacy and mechanistic properties of crude *Warburgia salutaris* leaf extract, both in its unencapsulated form (WSN) and when encapsulated within liposomal carriers (WSE). The integration of complementary analytical techniques—Fourier-transform infrared (FTIR) spectroscopy, powder X-ray diffraction (PXRD), and dynamic light scattering (DLS)—provided robust evidence for the successful encapsulation and compatibility of *W. salutaris* phytochemicals within the DPPC–cholesterol liposomal matrix. PXRD analysis revealed that the crude plant extracts are inherently amorphous, while the lipid carriers display semi-crystallinity. The encapsulated formulation retained both amorphous and semi-crystalline characteristics, indicating that the amorphous bioactive phytochemicals were stably integrated into the lipid matrix without significant alteration to their physical state. Such preservation of the amorphous phase is advantageous, as it can enhance the solubility and bioavailability of the encapsulated compounds.

FTIR spectroscopy further corroborated the successful encapsulation, showing spectral features characteristic of plant-derived functional groups, including O-H, ester C=O, P=O, and P-O-C. Subtle spectral shifts in these regions indicate the presence of non-covalent interactions, such as hydrogen bonding and van der Waals forces, between the phytochemicals and the lipid bilayer [57]. These interactions are likely to influence encapsulation efficiency, vesicle stability, and release kinetics, all of which are critical for the effective delivery of bioactive agents.

The DLS measurements demonstrated that the liposomal formulation exhibited an average particle size of 159.4 nm and a low polydispersity index (PDI = 0.1140), indicating a highly uniform population of nanoscale vesicles. Furthermore, the liposomes displayed a high positive zeta potential of +79.3 mV, reflecting strong electrostatic repulsion that prevents particle aggregation and supports long-term colloidal stability. These physicochemical attributes, together with the PXRD and FTIR findings, confirm that the liposomal formulation maintains both structural and chemical integrity, making it suitable for the efficient delivery of bioactive phytochemicals. The amorphous nature of the encapsulated compounds is of particular interest, as it is known to facilitate strong interactions with phospholipid headgroups, reduce crystallisation within vesicles, and improve both the stability and entrapment efficiency of bioactive molecules. These findings are consistent with previous studies that have highlighted the benefits of amorphous systems in enhancing the performance of liposomal drug delivery platforms.

In vitro cytotoxicity assays were performed to compare the potency of both WSN and WSE extracts on cellular metabolic activity on MCF-7 (human breast cancer) and Vero (African green monkey kidney epithelial) cell lines. IC_50_ values were determined for each extract, revealing a significant reduction in metabolic activity in MCF-7 cells for both WSN and WSE, with WSN exhibiting the lowest IC_50_. These results indicate that *W. salutaris* extracts, whether encapsulated or not, possess cytotoxic effects against cancer cells, as evidenced by reduced mitochondrial dehydrogenase activity in the MTT assay. In contrast, both Vero and RAW 264.7 (murine macrophage-like) cells showed increased metabolic activity upon treatment with WSN and WSE, suggesting selective cytotoxicity towards cancer cells. The observed differences in therapeutic response between cancerous and non-cancerous cell lines may be explained by the Warburg effect, which describes the preferential use of glycolysis over oxidative phosphorylation by cancer cells for ATP production [58]. The decline in MCF-7 cell metabolic activity could be attributed to various cytotoxic compounds identified in the LC-MS analysis of *W. salutaris*, including 3-prenylated flavans, naphthopyrans, lignans, naphthopyranones, linear diarylheptanoids, aurone flavonoids, biflavonoids, anthracenes, diterpenes, and lactones—all of which have demonstrated cytotoxic activities.

Additionally, both WSN and WSE extracts were found to inhibit cell proliferation in MCF-7 cells at a concentration of 0.3 mg/mL, indicating potential antimetastatic properties. This effect may be related to the presence of anthracenes, lignans, and phenylnaphthalenes, compounds known for their ability to inhibit cell proliferation and induce cytotoxicity in cancer models [59,60]. To further elucidate the cellular effects, nuclear staining assays using DAPI and Propidium Iodide (PI) were conducted. *W. salutaris*-treated cells showed positive staining with both stains DAPI and PI, indicating compromised nuclear integrity and chromatin disintegration. WSN treatment resulted in more intense fluorescence, suggesting that potent anti-tumour compounds such as naphthacenes, angucyclines, and diterpene lactones may induce apoptosis by allowing PI to access and stain nucleic material, producing a characteristic red fluorescence Chaundhry 2022. DAPI, on the other hand, readily binds to fragmented DNA, producing blue fluorescence and further confirming apoptotic activity.

Gene and protein expression analyses provided additional mechanistic insights. Quantitative PCR revealed that pro-apoptotic genes Bax and caspase-3 were upregulated in response to both WSN and WSE, while the anti-apoptotic gene Bcl-2 was downregulated. Protein assays confirmed the activation of key apoptotic mediators, including Bax, Bad, cleaved caspase-3, SMAC/Diablo, Trail R1/DR4, Trail R2/DR5, TNF RI/TNFRSF1A, Fas/CD95, FADD, and phosphorylated p53 (S15, S46), indicating the initiation of both intrinsic (mitochondrial) and extrinsic (death receptor) apoptotic pathways. While many cancer studies focus primarily on inducing cancer cell death, this work shifts attention to inflammation, an important component of the immune response that combats infections. However, studies of the cancer microenvironment have shown that inflammation can also stimulate cancer progression [61,62]. Therefore, compounds that possess both anticancer and anti-inflammatory properties are highly desirable [63]. This provides a rationale for the use of crude extracts rather than isolated compounds, as crude extracts may contain multiple bioactive constituents capable of targeting both processes simultaneously [64].

The anti-inflammatory properties of the extracts were evaluated through their effects on LPS-induced reactive oxygen species (ROS) production in RAW 264.7 macrophages. Both WSN and WSE demonstrated the ability to inhibit LPS-induced ROS generation, with WSE showing superior efficacy. This suggests that encapsulation not only preserves but may enhance the bioactivity of anti-inflammatory constituents. The inhibition of ROS production is likely due to the presence of compounds such as 2-arylbenzofuran flavonoids, linear diarylheptanoids, naphthopyrans, curcuminoids, aurone flavonoids, and hexacarboxylic acid derivatives, all of which have established anti-inflammatory activities [65,66]. Interestingly, at the lowest tested concentration (0.125 mg/mL), WSN was observed to directly induce cell death more than WSE. This could be attributed to the rapid release and direct interaction of unencapsulated bioactive compounds with cellular targets, leading to swift cell membrane damage and death. In contrast, the liposomal-encapsulated WSE afforded a more controlled and gradual activation of apoptotic pathways, highlighting the potential of encapsulation for modulating drug release and reducing off-target effects. Such controlled delivery is particularly desirable for minimising toxicity and side effects while maximising therapeutic efficacy in clinical applications.

Taken together, these results demonstrate that both crude and liposomal-encapsulated *W. salutaris* extracts possess significant anti-cancer, anti-proliferative, pro-apoptotic, and anti-inflammatory activities. Encapsulation within liposomal carriers enhances the stability, bioavailability, and controlled release of bioactive compounds, potentially reducing cytotoxicity to normal cells while maintaining or improving efficacy against cancer cells. These findings provide a strong foundation for further in vitro and in vivo studies aimed at optimising delivery systems, elucidating mechanisms of action, and ultimately developing novel therapeutic strategies based on *W. salutaris* phytochemicals.

## 4. Materials and Methods

*Warburgia salutaris* was selected based on ethnobotanical literature and its material collected in the gardens of Lowveld South African National Botanical Institute (SANBI), Mbombela, South Africa (25°27′57″ S, 30°59′07″ E). The plant specimens were then authenticated by a taxonomist in the Department of Biology and Environmental Sciences at the Sefako Makgatho Health Sciences University. The leaves and stems of the plant were dried at room temperature and ground into a fine powder using a Polymix PX-MFC 90 D high-frequency impact mill (Kinematica AG, Malters, Switzerland). Plant material extracts were prepared according to the method described Eloff, J. (1998) [29]. Briefly, approximately 20 g of each milled plant material was extracted with 200 mL of an equal mixture of dichloromethane and methanol (50:50) and shaken for 24 h at room temperature. The dichloromethane-methanol (50:50) solvent system was selected to enable efficient extraction of a broad spectrum of phytochemicals, including lipophilic terpenoids and moderately polar phenolic compounds. This solvent combination is widely used in phytochemical screening due to its ability to maximise compound recovery and biological activity. The extracts were then filtered through a Whatman No. 1 filter paper (Whatman No. 1, Boeco Qualitative filter, grade 3 hw 125 mm, BOECO Germany GmbH, Hamburg, Germany), followed by solvent evaporation using a rotary evaporator (Stuart RE300 Rotary Evaporator, Stuart, UK). The dried extracts were stored in airtight containers at 4 °C.

### 4.1. Synthesis of Liposomal Nanoparticles

Liposomes were manufactured using thin-film hydration, following the method of Umbarkar et al. (2021) [30], with minor modifications. In a clean round-bottom flask, 10 mL of methanol was used to dissolve phospholipids (dipalmitoyl phosphatidylcholine (DPPC), 100 mg) and cholesterol (10 mg, for membrane stability), along with the leaf extract of *W. salutaris* (10 mg). The mixture was placed in a rotary evaporator, and the solvent was removed under reduced pressure at ~45 °C. This formed a uniform, thin lipid layer on the inside surface of the flask. Subsequent vacuum drying removed any residual solvent. Ten millilitres of phosphate-buffered saline (PBS) was added to the lipid film. The temperature exceeded the phase transition point of DPPC (~50 °C), and the mixture was gently agitated to form multilamellar vesicles. Probe sonication was used for 5–10 min to reduce liposome size, producing small, uniform unilamellar vesicles. The liposomes were placed in sterile vials and stored at 4 °C until needed.

### 4.2. FTIR Spectroscopy

The FTIR spectra of the plant extracts, liposomal encapsulation, and liposomes were analysed using the Cary 630 Four Transform spectrophotometer (Agilent Technologies, Santa Clara, CA, USA) and subsequent analysis with OriginPro™ ver.2021-2022 (OriginLab Corporation, Northampton, MA, USA). An amount of 6 g of lyophilised sample was placed on a diamond crystal and examined within the wavenumber range of 400–4000 cm^−1^ at 2 mm/s, with a resolution of 4 cm^−1^ at room temperature. FTIR spectra were recorded for the samples analysed. The analysis enables the identification of key biomolecules and provides insight into interactions between plant-derived phytochemicals and phospholipid vesicles during liposomal encapsulation.

### 4.3. Powder X-Ray Diffraction

Powder X-ray diffraction pattern was employed using a D2 Phaser XE-T Edition instrument (Bruker, Billerica, MA, USA). The instrument used was a Bruker AXS diffractometer (D8 Venture/Quest) with CuKα radiation (λ = 1.54056 Å) over 20° ≤ 2θ ≤ 80°, operating at 40 kV and 40 mA. The divergent slit was set to 0.2 mm, and monochromator scanning was performed at 2°/min with a step size of 0.025 ° and a step time of 1 s. The lyophilised sample was positioned on the sample holder and aligned to ensure it fit completely. The average crystallite size of the sample was estimated using the Scherrer equation, which relates the broadening of the diffraction peaks to the size of coherently diffracting domains according to the expression:

0.9λ/d =Bcosθ

where λ is the wavelength of the X-ray radiation, B is the full width at half maximum of the diffraction peak (in radians), θ is the Bragg angle, and 0.9 is the shape factor (K) that accounts for crystallite morphology. This relation indicates that peak broadening (B) increases inversely with crystallite size (d), providing a means to estimate nanoscale domain dimensions from PXRD data.

### 4.4. Zeta Potential

The formulated liposomes loaded with plant extracts were analysed using Nano-wave II Zeta-sizer (Microtrac, York, PA, USA). Ten millilitres of the liposomal encapsulated extract was diluted with 100 mL of distilled water before analysis, followed by ultrasonication to achieve homogeneity. The sample was transferred into a 1.5 mL Eppendorf tube, and a sonication probe was introduced for 10 min at 25 °C.

### 4.5. Cell Culture

Breast cancer cells (MCF-7), kidney cells (Vero), and macrophages (RAW 264.7) were obtained from Cellonex (Pty) Ltd., Johannesburg, South Africa, and cultured separately in T75 flasks (Sigma-Aldrich (Pty) Ltd., Johannesburg, South Africa, an affiliate of Merck KgaA, Darmstadt, Germany). Dulbecco’s Modified Eagle Medium (DMEM) and Roswell Park Memorial Institute Medium (RPMI-1640) were used for cell maintenance (Sigma-Aldrich (Pty) Ltd., an affiliate of Merck KGaA). Each medium contained 10% foetal bovine serum (FBS), 1% penicillin-streptomycin antibiotic cocktail, and 5% L-glutamine (Sigma-Aldrich (Pty) Ltd., an affiliate of Merck KGaA). Cultures were maintained at 37 °C in incubators with 5% CO_2_ and high humidity. Culture medium was replaced every 2 to 3 days until cells reached approximately 80 to 90% confluence. A Nikon Eclipse TS100 light microscope (Nikon Corporation, Tokyo, Japan) was used to systematically assess cell morphology, viability, and mycoplasma infection status. In subsequent experiments, cells were seeded at a density of 1 × 10^5^ cells/mL into 96-well plates. The MCF-7 human breast cancer cell line was selected as a representative model for studying breast cancer proliferation and apoptosis due to its well-characterised responsiveness to chemotherapeutic agents. Vero kidney epithelial cells were included as a non-cancerous control to assess selective cytotoxicity and potential toxicity toward normal cells. The RAW 264.7 murine macrophage cell line was selected to evaluate inflammatory responses and reactive oxygen species production, as macrophages play a critical role in tumour-associated inflammation.

### 4.6. Cell Viability Assay

The MTT assay was used to evaluate cellular metabolic activity in selected cells following a method described by Monama et al. (2025) [24]. Cultured MCF-7, Vero and RAW 264.7 cells were seeded in 96-well plates at a density of 1 × 10^5^ cells/mL and allowed to adhere overnight under standard conditions. The cells were exposed to various concentrations of extracts, ranging from 0.3 to 0.0195 mg/mL. Doxorubicin and hydrogen peroxide served as positive controls, while untreated cells acted as negative controls. After a 24-h incubation at 37 °C, 20 μL of 5 mg/mL MTT reagent was added to each well. The plates were incubated for a further 4 h to allow mitochondrial dehydrogenases in viable cells to reduce MTT to purple formazan crystals. Subsequently, the medium was removed, and the cells were gently rinsed once with pre-warmed PBS (pH 7.4). Intracellular formazan was dissolved in acidic isopropanol, and absorbance was read at 570 nm using a GloMax^®^ Multi-detection system (Promega Corporation, Madison, WI, USA). Results are expressed as mean ± standard deviations of two independent triplicate experiments.

$$Percentage\;viability\;\left(\%\right)=\frac{Absorbance\;of\;treated\;cells}{Absorbance\;of\;untreated\;cells}\times 100$$

### 4.7. Anti-Metastatic Assay in MCF-7 Cells

The anti-metastatic activity of WSN and WSE leaf extracts was evaluated using an in vitro scratch assay. MCF-7 cells were cultured in RPMI medium supplemented with 10% fetal bovine serum (FBS), 5% L-glutamine, and 5% penicillin–streptomycin and incubated at 37 °C in a humidified atmosphere containing 5% CO_2_ until reaching approximately 70–80% confluency. A straight scratch was created across the cell monolayer using a sterile 10 µL pipette tip. The cells were gently washed with phosphate-buffered saline (PBS) to remove detached cells and debris. Cells were then treated with WSN and WSE extracts at a concentration of 0.3 mg/mL. Untreated cells served as the negative control, while cells treated with doxorubicin were used as the positive control. Images of the wound area were captured at 0 h and 24 h using an inverted microscope (Nikon Eclipse TS100) at 10× magnification. The same region of the scratch was imaged at both time points.

### 4.8. H2DCF-DA Assay

Reactive oxygen species (ROS) were evaluated in macrophage cells (RAW 264.7) using a modified protocol based on Chen et al. (2022) [60]. The cells were seeded at a density of 6 × 10^4^ cells per well in a 96-well plate and incubated at 37 °C in 5% CO_2_ for 24 h. After incubation, cells were treated with 100 μL of a 125 µg/mL dichloromethane: methanol (50:50) extract of *W. salutaris* and with lipopolysaccharide (LPS) (10 µg/mL) as a positive control. After incubation for 24 h, 10 µM H2DCF-DA was added and incubated in the dark for an additional 30 min. Fluorescence was read using a microplate reader at 485 nm (excitation) and 535 nm (emission). The RAW 264.7 cells were also cultivated on coverslips in 6-well plates and received identical treatments. The slides were then prepared for visualisation, and images showing the effect of LPS-induced ROS generation by extracts were captured using Zoe fluorescent microscopy (Bio-Rad Laboratories, Hercules, CA, USA) with the H2DCF-DA dye.

### 4.9. RNA Extraction and Purification

To evaluate the transcriptional response of MCF-7 breast cancer cells to extracts, total RNA was isolated following a 24-h treatment period at a concentration of 1000 μg/mL. The extraction process utilised the GeneJET RNA Purification Kit (Thermo Fisher Scientific, Johannesburg, South Africa) in accordance with the manufacturer’s instructions. Briefly, approximately 1.0 × 10^5^ cells were harvested via centrifugation at 1000 rpm for 5 min. The resulting cell pellets were washed with sterile phosphate-buffered saline (PBS) to eliminate residual culture media and centrifuged again. The washed cells were resuspended in 600 µL of lysis buffer supplemented with β-mercaptoethanol to stabilise the RNA. A 250 µL aliquot of this lysate was then homogenised with 360 µL of absolute ethanol. The mixture (700 µL) was loaded onto a GeneJET purification column and centrifuged at 12,000 rpm for 1 min. Contaminants were removed through a series of wash steps: first with 700 µL of Wash Buffer 1, followed by a 600 µL wash and a final 250 µL wash with Wash Buffer 2, each followed by centrifugation at 12,000 rpm. Finally, the purified RNA was eluted into a sterile tube using 100 µL of nuclease-free water and immediately processed for downstream applications.

### 4.10. First-Strand cDNA Synthesis

The purity and concentration of the isolated total RNA were quantified using a Qubit 2.0 Fluorometer (Invitrogen Life Technologies, Singapore). For the synthesis of complementary DNA (cDNA), 0.1–0.5 μg of total RNA served as the template. Reverse transcription was performed using the RevertAid First Strand cDNA Synthesis Kit (Thermo Scientific). The synthesis reaction was conducted in a total volume of 20 μL, comprising 1 μL of template RNA and a master mix of reagents. This mixture included Oligo(dT) primers, 5X reaction buffer, RiboLock RNase Inhibitor, 10 mM dNTP mix, and RevertAid M-MuLV Reverse Transcriptase. After gentle homogenization and brief centrifugation, the samples were incubated at 42 °C for 60 min to facilitate primer extension. The enzymatic reaction was subsequently terminated by thermal denaturation at 70 °C for 5 min. The resulting cDNA was stored at appropriate temperatures for subsequent PCR analysis.

### 4.11. Polymerase Chain Reaction (PCR)

Polymerase Chain Reaction (PCR) amplification was performed according to the protocol outlined by Monama et al. (2025) [24]. Reactions were performed on a QuantStudio™ 3 thermal cycler (Applied Biosystems, Thermo Fisher Scientific, Waltham, MA, USA). The cycling conditions included an initial denaturation at 95 °C for 10 min, followed by 30 cycles of denaturation at 95 °C for 30 s, primer annealing at 61 °C for 30 s, and extension at 72 °C for 60 s. A final extension step of 72 °C for 7 min was followed by a hold at 4 °C. PCR products were separated on a 1.5% agarose gel containing 0.5 µg/mL ethidium bromide, visualised with a ChemiDoc imaging system (Bio-Rad Laboratories (Pty) Ltd., Johannesburg, South Africa). Densitometric analysis quantified band intensities normalised to GAPDH expression, measured mRNA levels relative to other genes, and reported results as fold changes. Data analysis was performed using GraphPad Prism 8.4, and results were shown as mean fold increase ± SEM for gene expression percentages. The primer sequences used are listed in Table 3.

### 4.12. Annexin V-FITC and Propidium Iodide (PI), Analysis by Muse^®^ Cell Analyser

The quantification of apoptotic cell populations was conducted via flow cytometry, following the dual-staining protocol described by Esquivel-Campos, et al. (2022) [36]. The MCF-7 cell lines were exposed to different concentrations of EA/DCM fractioned leaf extracts of *W. salutaris* for four hours. After treatment, cells were harvested with a sterile cell scraper, centrifuged at 800 rpm for 10 min, and washed twice with 1 mL of ice-cold phosphate-buffered saline (PBS). The cells were then fixed in 4% paraformaldehyde at 4 °C overnight. For staining, cells were incubated in the dark at 37 °C for 30 min with 500 µL of a solution containing propidium iodide, RNase A, and buffer. Following incubation, cell viability and counting were performed using the Guava Muse^®^ Cell Analyzer (Luminex Corporation, Austin, TX, USA) to quantify apoptotic cells, cell death, and viable cells. Cells cultured in 6-well plates were used in a single experiment.

### 4.13. Human Apoptosis Proteome Array

The protein expression assay was prepared according to the manufacturer’s instructions for the Proteome Profiler Human Apoptosis Array Kit (Cat. No. ARY009, R&D Systems, Minneapolis, MN, USA), and the manufacturer’s instructions were adhered to throughout the process. Each well of a 4-well multi-dish was filled with 2 mL of Array Buffer 1, which served as the blocking buffer. The arrays were carefully removed from their protective sheets using flat-tip tweezers and placed into each well with the array number facing upwards. After incubation on a rocking platform shaker for one hour, cell lysates were diluted in Array Buffer 1, and the final volume was adjusted to 1.5 mL with lysis buffer. The maximum volume of each array was 250 µL. The blocking buffer was discarded, and samples were added to the wells. The plates were gently agitated and incubated overnight at 2–8 °C. The arrays were washed three times with 1× Wash Buffer, then incubated for 1 h with a diluted detection antibody cocktail. After several washes, the arrays were incubated in diluted Streptavidin-HRP for 30 min. After the final wash, the membranes were dried, and 1 mL of Chemi reagent mix was evenly applied and left for 1 min under plastic sheet protectors to ensure complete coverage. The excess reagent was wiped with absorbent wipes. The membranes, each labelled with its identifying number and facing up, were placed in an autoradiography cassette and covered with plastic. An X-ray film was used to expose the membranes for 1–10 min, with varying exposure times to enhance signal detection.

### 4.14. Liquid Chromatography-Mass Spectrometry

The phytochemical profile of the crude leaf extract of *W. salutaris* was analysed using a Waters Acquity UPLC system and a Synapt G2 QTOF mass spectrometer (Waters, Milford, MA, USA) with an electrospray ionisation source operated in negative mode. Approximately 50 mg of dried, powdered leaf material was mixed with 1 mL of 50% methanol containing 0.1% formic acid. The mixture was vortexed, sonicated for 1 h, and then centrifuged at 3000× *g* for 10 min. The supernatant was filtered through a 0.22 µm syringe filter. Each sample underwent three analyses. Separation was achieved on an Acquity BEH C18 column (2.1 × 100 mm, 1.7 µm) maintained at 55 °C, using a mobile phase of water (0.1% formic acid, A) and acetonitrile (0.1% formic acid, B) at a flow rate of 0.35 mL/min. The gradient programme was: 0–0.5 min, 100% A; 0.5–3 min, 22% B; 3–7 min, 44% B; 7–12 min, 100% B, held for 2 min, then re-equilibrated to initial conditions by 15 min. The injection volume was 3 µL, and the eluate was directed through a PDA detector before mass spectrometry analysis. Data were acquired in MSE mode from *m*/*z* 150 to 1500 by alternation between low energy (4 V) and high energy (40–100 V) to capture precursor and fragment ions. The source parameters included a capillary voltage of 2.5 kV, a cone voltage of 15 V, and a desolation gas flow rate of 650 L/h at 275 °C. Sodium formate served as the calibration standard, while leucine enkephalin was used as the lock mass. MS-DIAL software ver.5.5 as used to identify and align peaks in the raw data. Subsequently, MS-FINDER ver.3.6 was utilised to investigate the exact masses, isotopic patterns, retention characteristics, and in silico fragmentation of compounds in relation to metabolome databases. A catechin calibration curve (0.2–5 mg/L) was used for semi-quantification, and features that remained consistent across three injections were retained for further analysis.

### 4.15. Statistical Analysis

Statistical analysis was performed using GraphPad Prism version 8.4. All experimental results were expressed as means ± standard deviation (SD) from at least three independent experiments performed in triplicate, and significance was determined by one-way ANOVA with nonlinear regression for IC50 values (*p* < 0.05).

## 5. Conclusions

In conclusion, this study robustly demonstrates that crude leaf extracts of *Warburgia salutaris*, as well as their liposomal-encapsulated forms, exhibit pronounced anti-cancer, anti-tumour, anti-proliferative, and anti-inflammatory activities. The results underscore the potential of liposomal nanocarriers not only to enhance the stability and controlled release of bioactive compounds but also to improve selective cytotoxicity towards cancer cells while minimising adverse effects on normal cells. These findings align with and extend prior evidence supporting the therapeutic promise of amorphous, encapsulated phytochemicals in targeted drug delivery systems.

Importantly, the differential effects observed in cancerous versus non-cancerous cell lines and the significant inhibition of inflammatory responses collectively provide a comprehensive mechanistic rationale for further development of *W. salutaris*-based therapeutics. Moving forward, our research will systematically investigate both isolated bioactive constituents and liposomal bioencapsulated extracts in advanced in vitro and in vivo models, with a specific focus on their efficacy in reversing tumour progression and mitigating inflammation, particularly in murine systems. Additional studies are planned to characterise drug release kinetics and permeability, thereby enabling a detailed assessment of bioavailability and pharmacodynamic profiles under physiologically relevant conditions.

Altogether, these efforts will contribute critical insights into the optimisation of delivery platforms, the elucidation of molecular mechanisms, and the rational design of innovative phytochemical-based therapies. Ultimately, this work lays a strong foundation for the translational development of *W. salutaris* as a novel candidate for safe and effective cancer and inflammation management, with the potential to inform broader strategies in natural product drug discovery and nanomedicine.

## Figures and Tables

**Table 1:** Physicochemical Characterisation of Optimised *W. salutaris* leaf extracts-loaded liposomes.

Parameter	Value	Comments
Average Particle size (nm)	159.4	Nanoscale vesicle suitable for drug delivery
Polydispersity Index (PDI)	0.114	A narrow size distribution indicates a monodisperse vesicle population
Zeta potential (mV)	+79.3	Strong positive surface charge, strong electrostatic repulsion, enhances colloidal stability and prevents aggregation

**Table 2:** The LC-MS chromatogram of phytochemicals identified in the crude leaf extract of *Warburgia salutaris*, with metabolites eluting between 4.47 and 13.94 min.

ID	Retention Time (min)	Measured (*m*/*z*)	Compound Name	Biological Activity	Molecular Formula
545	5.4	507.14468	2-arylbenzofuran flavonoids	Antioxidant, Anti-inflammatory	C_31_H_24_O_7_
605	5.464	563.17297	3-prenylated flavans	Anticancer, Cytotoxic	C_34_H_28_O_8_
435	7.079	455.18509	Naphthacenes	Antimicrobial, Antitumor	C_29_H_28_O_5_
573	7.311	537.15637	Anthracenes	Cytotoxic, Antiproliferative	C_32_H_26_O_8_
315	7.473	405.22162	Linear diarylheptanoids	Antioxidant, Anti-inflammatory	C_30_H_30_O
327	7.761	413.17435	Naphthopyrans	Anti-inflammatory, Cytotoxic	C_27_H_26_O_4_
474	9.048	469.19977	Curcuminoids	Anticancer, Anti-inflammatory	C_30_H_30_O_5_
631	9.175	579.20422	Linear diarylheptanoids	Anti-inflammatory, Cytotoxic	C_35_H_32_O_8_
318	9.578	409.17892	Aurone flavonoids	Antioxidant, Anticancer	C_28_H_26_O_3_
572	9.802	531.14600	Aurone flavonoids	Anti-inflammatory, Cytotoxic	C_33_H_24_O_7_
409	9.893	445.17993	Phenylnaphthalenes	Antiproliferative, anti-cancer	C_31_H_26_O_3_
494	10.749	475.19086	Linear diarylheptanoids	Anticancer, Anti-inflammatory	C_32_H_28_O_4_
394	11.486	414.41967	Lignans	Anticancer, Cytotoxic	C_22_H_22_O_8_
538	11.585	501.17010	Biflavonoids	Antiproliferative, anti-cancer, Cytotoxic	C_33_H_26_O_5_
452	11.592	461.13800	Naphthopyranones	Cytotoxic, Anticancer	C_30_H_22_O_5_
544	11.592	507.14429	Anthracenes	Cytotoxic, anti-cancer, Antiproliferative	C_31_H_24_O_7_
758	11.684	755.21619	Hexacarboxylic acid derivatives	Anti-inflammatory, anti-cancer, Cytotoxic	C_37_H_40_O_17_
751	11.959	739.22125	Linear diarylheptanoids	Anticancer, Anti-inflammatory	C_44_H_36_O_11_
748	12.986	735.22589	Angucyclines	Antimicrobial, Antitumor	C_38_H_40_O_15_
640	13.126	585.24927	8-O-methylated flavonoids	Anticancer, Cytotoxic	C_35_H_38_O_8_
674	13.574	607.30713	Linear diarylheptanoids	Anticancer, Anti-inflammatory	C_39_H_44_O_6_
619	13.588	575.27985	Lignans	Antiproliferative, anti-cancer, Cytotoxic	C_38_H_40_O_5_
700	13.941	635.26794	Diterpene lactones	Cytotoxic, Antitumor	C_32_H_44_O_13_
246	13.377	341.10751	Sugars	Nutrient, not bioactive, anti-cancer	C_12_H_22_O_11_

**Table 3:** The primer sequences for gene amplification of GAPDH, *caspase-3*, *bax* and *bcl-2*.

Gene	Primer Sequence
*caspase-3*	Forward: 5 ′CCATGGGTAGCAGCCTCCTTC 3′ Reverse: 3 ′TGCGCTGCTCTGCCTTCT 5′
*Bax*	Forward: 5 ′TCCCCCCAGAGGTCTTTT 3′ Reverse: 3 ′CGGCCCCAGTTGAAGTTG 5′
*bcl-2*	Forward: 5 ′CTGCACCTGACGCCCTTCACC 3′ Reverse: 3 ′CACATGACCCCACCGAACTCAAAGA 5′
*GAPDH*	Forward: 5 ′TGCGCTGCTGCTCTGCCTTCT 3′ Reverse: 3 ′CCATGGGTAGCAGCTCCTTC 5′

## Data Availability

The original contributions presented in this study are included in the article. Further inquiries can be directed to the corresponding authors.

## Abbreviations

The following abbreviations are used in this manuscript:

Bax	Bcl-2-associated X protein
Bcl-2	B-cell lymphoma 2
DLS	Dynamic Light Scattering
FTIR	Fourier Transform Infrared Spectroscopy
H_2_DCF-DA	2′,7′-Dichlorodihydrofluorescein diacetate
IC_50_	Half-maximal inhibitory concentration
LC–MS	Liquid Chromatography–Mass Spectrometry
LPS	Lipopolysaccharide
PDI	Polydispersity Index
PXRD	Powder X-ray Diffraction
RT-PCR	Reverse Transcription Polymerase Chain Reaction
WSE	*Warburgia salutaris* liposomal-encapsulated crude leaf extract
WSN	*Warburgia salutaris* unencapsulated crude leaf extract
